# Clinical characteristics and prognostic factors of 60 patients with acquired immune deficiency syndrome combined with Cryptococcus neoformans

**DOI:** 10.1186/s12879-023-08137-8

**Published:** 2023-04-06

**Authors:** Saiduo Liu, Wei Chen, Fang Cheng, Xinchun Ye, Ning Pan, Hongzhou Lu

**Affiliations:** 1 Departments of Infectious Disease, The Dingli Clinical College of Wenzhou Medical University, Wenzhou, Zhejiang 325000 China; 2 Department of Infectious Disease, The 1st School of Medicine,School of Information and Engineering,The1st Affiliated Hospital of Wenzhou Medical University, Wenzhou, Zhejiang 325000 China; 3 Department of Radiology, The 2ed Affiliated Hospital of Wenzhou Medical University, Fuxue Lane, No. 2, Wenzhou, Zhejiang 325000 China

**Keywords:** Acquired immune deficiency syndrome, *Cryptococcus neoformans*, Meningitis, Prognosis, Clinical features

## Abstract

**Objective:**

Cryptococcal meningitis (CM) threatens people’s health and is the main cause of opportunistic fungus-related death in acquired immune deficiency syndrome (AIDS) patients. Herein, we investigate the clinical characteristics and prognostic factors of AIDS patients with *Cryptococcus neoformans* in Wenzhou, Zhejiang Province, China.

**Methods:**

Our study enrolled AIDS patients diagnosed with *Cryptococcus neoformans* infection who were hospitalised in our hospital. They were divided into Group A (32 patients with CM) and Group B (28 patients without CM) according to their diagnosis. The differences between the two groups of patients’ clinical symptoms, imaging examinations and laboratory examinations were observed. Statistical methods were used to analyse the difference in prognosis between the two groups.

**Results:**

Headache and fever were the most common clinical characteristics for patients with CM, while respiratory symptoms and fever were the most common clinical characteristics for patients without CM. The positive rate of cryptococcal capsular antigen, India ink staining and culture in the cerebrospinal fluid examination was higher in the CM patients than in the non-CM patients. The overall morbidity and mortality rate after systemic antifungal therapy was higher in the CM patients than in the non-CM patients. A higher incidence of headache, impaired consciousness, nuchal rigidity, first intracranial pressure > 200 mmH_2_O and mortality was observed in the CM patients than in the non-CM patients. Multifactorial logistic regression analysis showed that headache risk factors affecting the patient’s prognosis at 12 weeks.

**Conclusion:**

Patients with AIDS diagnosed with *Cryptococcus neoformans* infection have insidious clinical symptoms in the early stage, and their manifestation is often non-specific, resulting in poor prognosis and high mortality among CM patients compared to patients without CM. Therefore, early identification and timely antifungal therapy before the disease progresses to meningitis are of great value in improving the survival rate of patients.

## Introduction

The first official report on acquired immune deficiency syndrome (AIDS) was published in the *Morbidity and Mortality Weekly Report* on 5 June 1981 in the United States. Acquired immune deficiency syndrome is caused by human immunodeficiency virus (HIV) infection and is one of the most serious public health problems worldwide. The Joint United Nations Programme on HIV and AIDS reported that by the end of 2020, there were 37.7 million living people with HIV/AIDS worldwide, and about 1.5 million new HIV infections are detected each year [1].

In China, AIDS is a serious threat to the health of the population, and the main cause of death is an opportunistic infection [2]. According to ‘Progress in AIDS Epidemiology in China’, by the end of 2020, approximately 1.05 million people were living with HIV in China, with a cumulative death toll of 350,000. *Cryptococcus neoformans*, one of the conditional pathogens causing opportunistic infections in humans, is the leading cause of death in patients with advanced AIDS, with the highest incidence of infection in sub-Saharan Africa [3, 4]. Cryptococcal meningitis (CM) is a deep fungal disease caused by a new type of cryptococcal infection. In AIDS patients, HIV is prone to violate the blood–brain barrier, which facilitates the spread of *Cryptococcus neoformans* pathogens to the central nervous system, causing CM [5, 6]. Recently, the World Health Organization (WHO) issued the first fungal priority pathogen list for invasive fungal diseases, classifying *Cryptococcus neoformans* as a serious priority pathogen. The literature reports that 15–20% of AIDS-related deaths are caused by CM, and the mortality rate of AIDS combined with CM is reported to be 20–40% in different regions and even up to 70% per year in resource-limited areas [3, 7]. The number of CM cases in China has also gradually increased over the past 20 years. Cryptococcal infection can present as asymptomatic pulmonary colonisation or cause severe central nervous system infection or systemic disseminated infection [8].

The purpose of this paper is to retrospectively analyse the clinical manifestations and laboratory data of AIDS combined with *Cryptococcus neoformans* infection, to explore the relevant factors affecting patient prognosis and to observe some clinical characteristics and changes in laboratory indicators at the time of initial diagnosis and after treatment. Moreover, we aim to provide a scientific basis for the early clinical diagnosis and treatment of AIDS combined with *Cryptococcus neoformans* infection, which would help further improve clinicians’ diagnosis and treatment of this disease.

## Research participants and study methods

### Research participants

Patients with AIDS combined with *Cryptococcus neoformans* infection who were hospitalised from March 2014 to October 2021 in the Department of Infection, Wenzhou Central Hospital, Wenzhou, Zhejiang Province, China, were selected as study participants. The study was approved by the hospital’s ethics committee. The inclusion criteria of the study participants were as follows: (1) age ≥ 18 years; (2) all AIDS patients met the relevant regulations in the ‘AIDS Treatment Guidelines’ (2020 edition) [9]; and (3) all AIDS patients met the diagnosis of *Cryptococcus neoformans* infection. The exclusion criteria of the study participants were as follows: (1) age < 18 years; (2) advanced combined malignancy; (3) asymptomatic patients with simple cryptococcal capsular antigen positivity; (4) combined with bacteria other than *Cryptococcus neoformans*; and (5) fatal severe infections. Ultimately, 60 participants were included in the study.

### Methods

#### Information collection

To conduct a comprehensive analysis, a retrospective study was conducted to collect basic information, epidemiological features, clinical manifestations, ancillary tests, treatment protocols and prognostic follow ups of patients. The diagnosis of AIDS combined with *Cryptococcus neoformans* was based on the following: (1) positive serological cryptococcal capsular antigen test results, and (2) patients with clear foci of infection, clinical symptoms and signs or abnormal laboratory test results.

The diagnosis of all CM patients was conducted according to the ‘Expert Consensus on the Clinical Management of AIDS Combined with Cryptococcosis’ [10]. The diagnostic criteria were as follows: (1) patients with AIDS who had meningitis-related symptoms, signs, abnormal cerebrospinal fluid examination results or abnormal cranial imaging; (2) *Cryptococcus* found by microscopic examination of cerebrospinal fluid India ink staining; (3) positive blood or cerebrospinal fluid cryptococcal culture; (4) positive blood or cerebrospinal fluid cryptococcal capsular antigen; (5) positive blood or cerebrospinal fluid cryptococcal antibodies or nucleic acid; and (6) *Cryptococcus* found by histopathological examination; any of condition met the first and (2)–(6) above can be diagnosed.

The prognostic criteria for AIDS combined with CM were as follows [11]: (1) Clinically cured – after treatment, the patient’s clinical symptoms disappeared, cerebrospinal fluid pressure and routine biochemical examination results returned to normal, no *Cryptococcus* was found in three consecutive cerebrospinal fluid, blood and bone marrow fungal cultures (every 7 d) and the patient was stable for more than 4 weeks; (2) Improved – clinical symptoms and signs improved significantly, cerebrospinal fluid pressure was normal and the results of the cerebrospinal fluid biochemical examination and bacterial culture were occasionally positive; and (3) Ineffective – after treatment, the patient’s clinical symptoms and signs did not improve significantly or worsened and the patient was either discharged or died. The 60 patients in the study were divided into Group A (32 patients with CM) and Group B (28 patients without CM).

#### Laboratory tests

All patient AIDS diagnoses were confirmed by the Wenzhou Center for Disease Control, and the laboratory confirmed HIV antibody positivity by immunoblotting, and all met the diagnostic criteria of the third edition of the AIDS treatment guidelines (2020 edition) [10]. The diagnosis of cryptococcosis was made with pathogenic evidence, including immunochromatography for blood cryptococcal capsular antigen, blood culture, cerebrospinal fluid India ink staining and cerebrospinal fluid examination from the hospital laboratory department. Routine, biochemical and microbiological cultures of cerebrospinal fluid, CD4 + cell count and other viral tests were also tested by the laboratory department.

### Statistical analysis

Data were analysed using SPSS Statistics 22.0 for Windows (IBM Corporation, Armonk, NY), and factors related to prognosis were analysed using a t-test for the comparison of means for normal measures and the Chi-square test or Fisher’s exact test for the comparison of rates of count data. Logistic regression was used to analyse patients’ 12-week prognostic risk factors, and the 13 factors of age, sex, time of HIV diagnosis, baseline CD4 + cell count, headache, impaired consciousness, nuchal rigidity, first intracranial pressure > 200 mmH_2_O, cerebrospinal fluid white blood cell count, cerebrospinal fluid protein, cerebrospinal fluid glucose, cerebrospinal fluid chloride and mortality were used as independent variables with P < 0.05 indicating statistical significance.

## Results

### General and epidemiological data

A total of 60 patients with AIDS combined with cryptococcosis, including 54 males (90.0%) and 6 females (10.0%), with an average age of 44.69 years, met the enrolment criteria. The route of HIV infection was clearly defined as homosexual transmission in 15 cases (25.0%), heterosexual transmission in 37 cases (61.6%), intravenous drug use in 1 case (1.7%) and in 7 cases (11.7%), the route of infection was not clearly defined. The duration of confirmed AIDS at the time of admission was 0–8 years. A total of 53 patients (88.3%) were confirmed positive for HIV antibodies after the first diagnosis with no treatment with highly active anti-retroviral therapy (HAART) or had discontinued HAART on their own before hospitalisation; 7 patients (11.9%) were treated with regular HAART before hospitalisation. Only 1 patient (1.7%) was not receiving treatment after admission to the hospital, and the other 59 patients (98.3%) were treated with AMB/L-AMB + 5-FC + FCZ/VRC (9 patients, 15%), AMB/L-AMB + FCZ/VRC (11 patients, 18.3%), AMB/L-AMB + 5-FC (8 patients, 13.3%), 5-FC + FCZ/VRC (8 patients, 13.3%), AMB/L-AMB (10 patients, 16.7%) and FCZ/VRC (13 patients, 21.7%).

### Clinical characteristics of patients with AIDS combined with cryptococcosis

In all patients with AIDS combined with cryptococcosis, 45 patients (75.0%) had a cough as the main manifestation, 29 patients (48.3%) had a fatigue, 26 patients (43.3%) had a headache, 20 patients (33.3%) had nuchal rigidity, 16 patients (26.6%) had vomiting, 8 patients (13.3%) had baseline CD4 + T-lymphocyte count above 100 cells/µl. As to other opportunistic infection, 31 patients (51.7%) had Other fungal infections, 14 patients (23.3%) had tuberculosis and 3 patients (5%) had Kaposi’s sarcoma, while 12 patients (20%) only had HIV combined with cryptococcal infection, as detailed in Table 1. A total number of 20 case (69.0%) had intracranial pressure above 200 mmH_2_O for Group A and 3 case (16.7%) for Group B. While a similar total number of 23 cases (71.9%) and 25 cases (89.3%) had blood Positive cryptococcal antigen between Group A and Group B. And a total number of 11 cases (34.4%) and 2 cases (7.1%) had blood Positive cryptococcal culture between Group A and Group B. 21 cases (35.0%) had positive ink staining of the cerebrospinal fluid and 3 cases had bone marrow aspiration, all of which were negative. Other details are shown in Table 2.

**Table 1:** Common clinical features of patients with AIDS combined with cryptococcus neoformans

Clinical features		Number of cases (n = 60)	Composition ratio (%)
Gender	Male	54	90.0%
	Female	6	10.0%
Signs and Symptoms	Fever	42	70.0%
	Headaches	26	43.3%
	Disorders of consciousness	9	15.0%
	Cough	45	75.0%
	Fatigue	29	48.3%
	Nuchal rigidity	20	33.3%
	vomiting	16	26.6%
CD4 + cell count	>100cells/µl	8	13.3%
	< 100cells/µl	52	86.7%
HARRT treatment	Conducted	7	11.7%
	None	53	88.3%
Opportunistic infection	Tuberculosis	14	23.3%
	Other fungal infections	31	51.7%
	Kaposi’s sarcoma	3	5.0%
	None	12	20.0%

**Table 2:** Results of intracranial pressure and cerebrospinal fluid examination in patients with AIDS combined with cryptococcus neoformans

	Projects	Group A	Group B
Intracranial pressure	>200	69.0% (20 cases)	16.7% (3 cases)
	< 200	31.0% (9 cases)	83.3% (15 cases)
Blood	Positive cryptococcal antigen	71.9% (23 cases)	89.3% (25 cases)
	Positive cryptococcal culture	34.4% (11 cases)	7.1% (2 cases)
Cerebrospinal fluid	Ink dyeing	72.4% (21 cases)	0
	Positive cryptococcal antigen	79.3% (23 cases)	0
	Positive cryptococcal culture	75.9% (22 cases)	0
Bone Marrow	Bone marrow aspiration	0.0% (0 in 3case)	0

Note: Three patients in group A did not undergo lumbar puncture due to their condition, 10 patients in group B refused lumbar puncture, three patients did not complete blood cryptococcal antigen testing, and only five patients completed blood cryptococcal culture testing

### Imaging features of patients with AIDS combined with meningitis

In Group A, 7 patients (21.9%) did not undergo imaging, and the remaining 25 patients (78.1%) underwent cranial magnetic resonance imaging or computed tomography, and the results showed 11 (44.0%) abnormalities, including 4 (16.0%) intracranial abnormal signal shadows distributed in the thalamus, parietal lobe and basal ganglia; 2 (8.0%) temporal lobe hypodense lesions; 1 (4.0%) cerebellar hemisphere abnormal enhancement with oedema; 2 (8.0%) basal ganglia semioval ischemic lesions; and 2 (8.0%) basal ganglia and temporal lobe lacunar infarcts. The images from the remaining 14 patients were normal (56.0%) (see Table 3).

**Table 3:** Imaging features of patients with AIDS combined with CM

	n = 32	Whether abnormal	Imaging Description
CT or MR cranial examination	78.1% (25 cases)	44.4% (11 cases abnormal)	Four cases had abnormal intracranial signals in the thalamus, parietal lobe and basal ganglia; two cases had hypointense foci in the temporal lobe; one case had abnormal enhancement of the cerebellar hemisphere with edema; two cases had ischemic foci in the basal ganglia and hemi-oval area and two cases had basal ganglia and temporal lobe lacunar infarcts.
		56% (14 cases normal)	-
No imaging was performed	21.9% (7 cases)	-	-

Note: CM, Cryptococcal meningitis; CT, Computed Tomography; MRI, Magnetic Resonance Image

### Treatment outcome and follow up

Among Group A, 1 case (3.1%) was misdiagnosed as viral meningitis and 2 cases (6.3) were misdiagnosed as tuberculous meningitis, 18 cases (56.3%) improved and 14 cases (43.8%) had a poor prognosis (6 deaths and 8 automatic discharges), and the cause of death was cryptococcal meningitis. In Group B, 3 cases (10.7%) had a poor prognosis (1 death and 2 automatic discharges), and 25 cases (89.3%) improved (see Table 4).

**Table 4:** Treatment outcome

Group	Improved	Poor prognosis	
Group A (n = 32)	56.3% (18 cases)	18.8% (6 cases)	Death
		25.0% (8 cases)	Automatic Discharge
Group B (n = 28)	89.3% (25 cases)	3.6% (1 case)	Death
		7.1% (2 cases)	Automatic Discharge

### Univariate analysis of 12-week prognosis in patients with AIDS combined with cryptococcosis

Univariate analysis of factors affecting the 12-week prognosis of the two groups showed that the incidence of headache, impaired consciousness, nuchal rigidity and mortality were statistically higher in Group A than in Group B (*P* < 0.05). When comparing the first intracranial pressure elevation and the cerebrospinal fluid India ink staining, cerebrospinal fluid culture results, cerebrospinal fluid white blood cell count, cerebrospinal fluid protein level, baseline CD4 + cell count, CD4/CD8, age, days in the hospital, duration of HIV diagnosis and cerebrospinal fluid glucose level between the two groups, the AIDS combined with CM group had a high positive rate with statistical differences (P < 0.05). In contrast, there was no statistical difference between the two groups for sex, and cerebrospinal fluid chloride. (see Table 5)

**Table 5:** Single factor affecting prognosis

influence factor	OR value (95% CI)	P value
age	1.05 (1.01,1.09)	0.009
sex	1.16 (0.21,6.27)	0.863
headache	0.01 (0,0.09)	< 0.001
impaired consciousness	0 (0,Inf)	< 0.001
nuchal rigidity	0.02 (0,0.2)	< 0.001
first intracranial pressure elevation (median [IQR])	0.98 (0.96,0.99)	< 0.001
baseline CD4 + cell count (median [IQR])	1.01 (1,1.02)	0.014
CD4/CD8 (median [IQR])	327.06 (1.63,65714.27)	0.003
mortality = 1 (%)	0 (0,Inf)	< 0.001
cerebrospinal fluid India ink staining	0 (0,Inf)	< 0.001
cerebrospinal fluid culture	0 (0,Inf)	< 0.001
days in the hospital	0.93 (0.88,0.97)	< 0.001
days of HIV diagnosis (median [IQR])	0.99 (0.96,1.01)	0.18
cerebrospinal fluid white blood cell count (median [IQR])	0.69 (0.45,1.04)	< 0.001
cerebrospinal fluid protein level (median [IQR])	0 (0,0.09)	< 0.001
cerebrospinal fluid chloride	1.13 (0.99,1.29)	0.052

Note: OR, odd ratio

### Multifactorial analysis affecting prognosis

Further incorporation of the above factors affecting prognosis into a multifactorial logistic regression analysis showed that headache was risk factors affecting the patient’s prognosis at 12 weeks (see Table 6).

**Table 6:** Multifactorial factor affecting prognosis

influence factor	OR value	95%CI	P value
headache	0.03	0.00-0.18	0.002
Hyperactivity of neck	0.09	0.00-1.15	0.075
Respiratory symptoms	2.94	0.47–18.87	0.235
Age	1.11	0.99–1.30	0.1
Intracranial pressure	1.01	1.00-1.02	0.124
CSF protein	0.07	0.00- 0.68	0.081

Note: OR, odd ratio

## Discussion

Cryptococcosis is a subacute or chronic deep fungal disease caused by *Cryptococcus neoformans* infection, which is widely distributed in nature and usually enters the body via the respiratory tract. *Cryptococcus neoformans* is one of the conditionally opportunistic pathogens, mostly occurring in immunocompromised patients with multiple and severe symptoms, which can present as a high fever, shortness of breath and other manifestations, with easily disseminated lesions and a high mortality rate [12, 13].

Among the 60 patients in this study, 52 had CD4 + lymphocyte counts below 100 cells/µl, which is consistent with clinical reports [14, 15]. Of these patients, only 8 cases were treated with formal antiviral therapy, and 52 cases were not treated with HAART or interrupted antiviral therapy at the time of consultation, which could suggest that patients with low CD4 + lymphocyte counts and not treated with HAART may have a poor prognosis. However, because of the small number of cases in this study, further research is needed on CD4 + lymphocyte maintenance levels, the timing of HAART treatment and protocol composition and prognosis.

All cryptococcosis patients in this study were diagnosed by detecting capsular antigen, smear and culture. The sensitivity and specificity of cryptococcal antigen (Cr-Ag) in serum and cerebrospinal fluid are reported to be as high as 95% in the literature [16], and Cr-Ag can diagnose infection on average three weeks earlier than CM symptoms or smears. Unfortunately, all patients in this study had Cr-Ag tests performed after the onset of symptoms, and routine follow-up Cr-Ag tests were not carried out for AIDS patients with initial negative cerebrospinal fluid cryptococcal capsular antigen, so data could not be obtained to confirm this. The positive rates of cerebrospinal fluid cryptococcal capsular antigen, cryptococcal ink staining and cryptococcal culture in Group A patients were 79.3%, 72.4% and 75.9%, respectively. The positive rates of blood cryptococcal capsular antigen and blood cryptococcal culture in Group B were 89.3% and 7.1%, respectively, which were slightly lower than the positive rates reported in the literature. The significance of Cr-Ag screening has been clearly stated in the guidelines of the WHO and China [9, 17], which suggest that serum and cerebrospinal fluid Cr-Ag screening should be enhanced in the late stage of AIDS, especially for CD4 + lymphocyte counts < 100 cells/µl. Several studies [14, 15] have shown that routine screening for Cr-Ag in AIDS patients with CD4 + lymphocyte counts < 100 cells/µl and interventions for antigen-positive patients can reduce the morbidity and mortality of CM. In this group, 68.33% of the patients were newly diagnosed as HIV positive, so HIV publicity should be improved to raise awareness of HIV in the general population, and CD4 + T-lymphocyte count screening and targeted Cr-Ag screening should be carried out in the HIV population.

Related research shows that the proportion of newly reported late-detection HIV/AIDS cases in medical institutions in China is more than 65% annually, and the number of late-detection cases is increasing year on year [18, 19]. The data from this study showed that most patients were confirmed to be HIV-positive after their first visit with a fever or headache and were already in the advanced stage of AIDS at the time of admission. Therefore, in patients with advanced AIDS, blood cryptococcal antigen should be tested as one of the means to screen for AIDS-combined cryptococcosis, especially when AIDS patients present with a fever, headache, cough and sputum; doctors should be highly alert to the possibility of combined cryptococcosis. In the related literature, it has been reported that AIDS combined with cryptococcosis has a high mortality rate in patients with combined CM, which is one of the main causes of death in AIDS patients [20]. The WHO estimates that CM accounts for 15% of all HIV-related deaths [21]. The overall mortality rate in our group of patients with AIDS combined with CM after systemic antifungal therapy was 18.8% (6/32), which is similar to that reported in the literature. The literature [22] reports that a low CD4 + T-cell count was a high-risk factor affecting the prognosis of patients with AIDS combined with CM, and the CD4 + T-lymphocyte count of the 32 patients in Group A was less than 100 cells/µl, accounting for 93.8%, which is consistent with clinical reports. Recently, the WHO released its updated ‘Guidelines for the Diagnosis, Prevention and Management of Cryptococcosis in adults, Adolescents and Children Living with HIV’. A single high dose of liposomal amphotericin B is strongly recommended in this update as a component of the preferred induction regimen for the treatment of CM in HIV-infected patients. The guidelines also note that this simplified treatment regimen is similar in effectiveness to the previously recommended regimen, with the advantages of lower toxicity and fewer monitoring needs. However, our study occurred before the publication of these guidelines, and we will look at the effectiveness of the new guideline therapy in a later study.

This study was a single-centre retrospective cohort study with some limitations. Most of the patients seen were in stage IV of the disease, often with other opportunistic infections, and the prognostic impact of these infections was not evaluated. Furthermore, the hospital admissions were mostly referred to AIDS patients, which may have some selection bias. In addition, this study did not analyse all patients with cryptococcal strains for drug resistance during treatment, which may have an impact on the assessment of the treatment effect. It is also necessary to further expand the number of observation cases and strengthen follow-up monitoring in future clinical work.

## Conclusion

After the development of meningitis, AIDS patients infected with *Cryptococcus neoformans* had a poor prognosis and high mortality. While the clinical symptoms are often non-specific in the early stage, resulting in poor prognosis and high mortality among CM patients compared to patients without CM. Therefore, the prevention and treatment of meningitis are of great value in improving the survival rate of patients. This means that prompt antifungal treatment to prevent meningitis before the infection progresses to impaired consciousness, first intracranial pressure > 200 mmH_2_O and nuchal rigidity is key to reducing the poor prognosis. And a lumbar puncture should be performed promptly to collect cerebrospinal fluid for pathogenetic testing, which together with cryptococcal capsular polysaccharide antigen and titer testing, are key to the prevention of meningitis and reducing the morbidity and mortality of CM.

## Data Availability

All data generated or analyzed during this study are included in this published article.
